# Electroactive Artificial Muscles Based on Functionally Antagonistic Core–Shell Polymer Electrolyte Derived from PS‐*b*‐PSS Block Copolymer

**DOI:** 10.1002/advs.201801196

**Published:** 2018-12-13

**Authors:** Van Hiep Nguyen, Jaehwan Kim, Rassoul Tabassian, Moumita Kotal, Kiwoo Jun, Jung‐Hwan Oh, Ji‐Myeong Son, Muhammad Taha Manzoor, Kwang Jin Kim, Il‐Kwon Oh

**Affiliations:** ^1^ Creative Research Initiative Center for Functionally Antagonistic Nano‐Engineering Department of Mechanical Engineering Korea Advanced Institute of Science and Technology (KAIST) 291 Daehak‐ro Yuseong‐gu Daejeon 34141 Republic of Korea; ^2^ Active Materials and Smart Living Laboratory Department of Mechanical Engineering University of Nevada Las Vegas (UNLV) Las Vegas NV 89154 USA

**Keywords:** block copolymers, ionic polymer actuators, polymer electrolytes, soft actuators, soft robotics

## Abstract

Electroactive ionic soft actuators, a type of artificial muscles containing a polymer electrolyte membrane sandwiched between two electrodes, have been intensively investigated owing to their potential applications to bioinspired soft robotics, wearable electronics, and active biomedical devices. However, the design and synthesis of an efficient polymer electrolyte suitable for ion migration have been major challenges in developing high‐performance ionic soft actuators. Herein, a highly bendable ionic soft actuator based on an unprecedented block copolymer is reported, i.e., polystyrene‐*b*‐poly(1‐ethyl‐3‐methylimidazolium‐4‐styrenesulfonate) (PS‐*b*‐PSS‐EMIm), with a functionally antagonistic core–shell architecture that is specifically designed as an ionic exchangeable polymer electrolyte. The corresponding actuator shows exceptionally good actuation performance, with a high displacement of 8.22 mm at an ultralow voltage of 0.5 V, a fast rise time of 5 s, and excellent durability over 14 000 cycles. It is envisaged that the development of this high‐performance ionic soft actuator could contribute to the progress toward the realization of the aforementioned applications. Furthermore, the procedure described herein can also be applied for developing novel polymer electrolytes related to solid‐state lithium batteries and fuel cells.

Soft robotics, which is a rapidly growing subfield of robotics mainly constructed with highly compliant materials, allows for accomplishing complex and delicate tasks with increased adaptability and improved safety, similar to living organisms. Therefore, developing high‐performance soft actuators and artificial muscles became a key challenging issue to realize such soft robots that can be practically implemented in engineering and industrial applications. Among several soft actuators listed in Table S1 in the Supporting Information, electroactive polymer (EAPs) actuators would have greater potential in comparison to the other counterparts since their motions are directly activated and accurately controlled by electrical signals as described in Table S2 in the Supporting Information. More specifically, ionic soft actuators have valuable and remarkable properties including the ability to exhibit large bending deformations at very low voltages (<5 V), space‐saving biomimetic operation, low noise and vibration, and low power consumption. Moreover, despite their infancy, such wet‐type ionic actuators were already used in some aquatic soft robots.^1^ Ionic actuators based on conducting polymers have relatively large stresses and strains, but usually operate in an electrolyte solution, which prevents open‐air applications.^2^ In contrast, dry‐type electroactive ionic actuators that comprise a polymer electrolyte membrane, an ionic liquid, and two conductive electrodes can perform in ambient air atmosphere, which would be great benefits to soft robotics operated in open‐air condition. In order to realize these applications, such actuators should have large and fast deformations under low voltages without back relaxation, along with long‐term durability. To obtain such excellent performances, the advances in both polymer electrolyte and electrode materials are required. However, while electrode materials such as carbon nanotubes, graphenes, and conducting polymers have been rigorously investigated, noble ionic exchangeable polymers that are specially designed for high‐performance ionic soft actuators have not been extensively developed.^3^, ^4^, ^5^, ^6^, ^7^, ^8^, ^9^

The polymer electrolytes play a key role in ion migration and mechanical stability in ionic soft actuators. However, developing excellent polymer electrolytes that have antagonistic properties between ionic conductivity and mechanical stability is a challenging issue since these two properties have an inversely interrelated relationship.^10^ In addition, although continuous ionic channels are desirable for efficient ion movement through open pathways, controlling such morphology over a thick film (>100 µm) could be difficult. For example, although Nafion, a commercially available polymer (Figure S1a, Supporting Information), is mainly considered as the most common and important ionic polymer in developing the ionic soft actuators, it suffers from the natural uncontrollability in the pattern of ionic channels due to the random distribution of the sulfonate groups. As a result, such pattern containing blocked ionic channels could be a reason for back relaxation phenomenon, which could induce inaccuracy and unreliability while applying to precise control systems and soft robots.^4^, ^5^, ^11^ Another commercial pentablock copolymer, poly[(*t*‐butyl‐styrene)‐*b*‐(ethylene‐*r*‐propylene)‐*b*‐(styrene‐*r*‐styrene sulfonate)‐*b*‐(ethylene‐*r*‐propylene)‐*b*‐(*t*‐butyl‐styrene)] (Figure S1b, Supporting Information), has the same issue despite its self‐assembly property. Although nanoadditives like sulfonated montmorillonite were introduced in an attempt to connect these separated conducting domains in order to facilitate ion movement, the actuation performances of those actuators could not be significantly improved, and their rise time was very long, requiring about 600 s.^6^ This issue was addressed by simplifying the pentablock copolymer through the elimination of the poly(*tert*‐butylstyrene) block, which yielded the diblock copolymer poly[(styrene‐*r*‐styrene sulfonate)‐*b*‐(ethylene‐*r*‐propylene)] or simply poly(styrenesulfonate‐*b*‐methyl butylene) (PSS‐*b*‐PMB) (Figure S1c, Supporting Information). As a result of this change in the architecture of the polymer, a continuous conducting phase was formed with the concomitant increase in the ionic conductivity. Therefore, the rise time of the actuator was reduced by ten times to 60 s. Notwithstanding, the nonconducting styrene units of the poly(styrene‐*co*‐styrene sulfonate) conducting phase could interfere with ion migration and the tiny mobile counterion (H^+^) could contribute modestly to deformation.^7^ Therefore, for the development of high‐performance soft actuators, the synthesis of new ionic exchangeable polymers is inevitably crucial.

Here, we report an unprecedented ionic exchangeable block copolymer electrolyte, PS‐*b*‐PSS‐EMIm, with a functionally core–shell architecture that was specifically designed for use in the ionic soft actuators. The PS‐*b*‐PSS‐EMIm was designed on the basis of the requirements of the actuators as the different properties of its distinct blocks could simultaneously afford the aforementioned antagonistic functionalities, and its self‐assembly ability could allow to control the pattern of ionic nanochannels (**Figure** **1** and Figure S1d, Supporting Information).^5^, ^6^, ^7^, ^12^ The conducting block, PSS‐EMIm, entirely comprises strong acid styrenesulfonate unit to facilitate ion movements. Such conducting block also contains adequate‐size mobile counterion, EMIm, to enhance actuation deformation. In general, small mobile counterions have high mobility, which produces much faster responses, but a modest contribution to the volume difference, leading to small bending. In contrast, big mobile counterions contribute significantly to the volume difference, resulting in large bending, whereas their low mobility causes much slower responses and larger phase delays.^13^ On the other hand, the structural block, PS with high glass transition temperature (*T*_g_ = 100 °C), provides adequate mechanical stiffness and strength to overall system for easy fabrication and the realization of practical applications. In this context, weak ionic polymers can hardly maintain their shape unaltered, eventually resulting in low blocking force, whereas hard polymers hamper electro‐chemo‐mechanical deformations. Furthermore, for an enhancement of the mechanical stability, block copolymers with high molecular weight are preferable.^5^, ^12^ To control the pattern of ionic nanochannels, the sphere morphology, in which PS spheres distribute in continuous PSS‐EMIm matrix, was selected for maximizing ion speed in short paths while providing good mechanical stability. Such morphology with high volume fraction of the conducting block could also favor the bending as a result of the high mobile counterion content. In addition, this specific self‐assembly would allow increasing both the antagonistic properties simultaneously. Thus, the conductivity could be enhanced by exclusively plasticizing the conducting phase through the addition of an ionic liquid, while the mechanical stability could be reinforced by selectively cross‐linking the mechanically structural phase.^14^, ^15^

**Figure 1:**
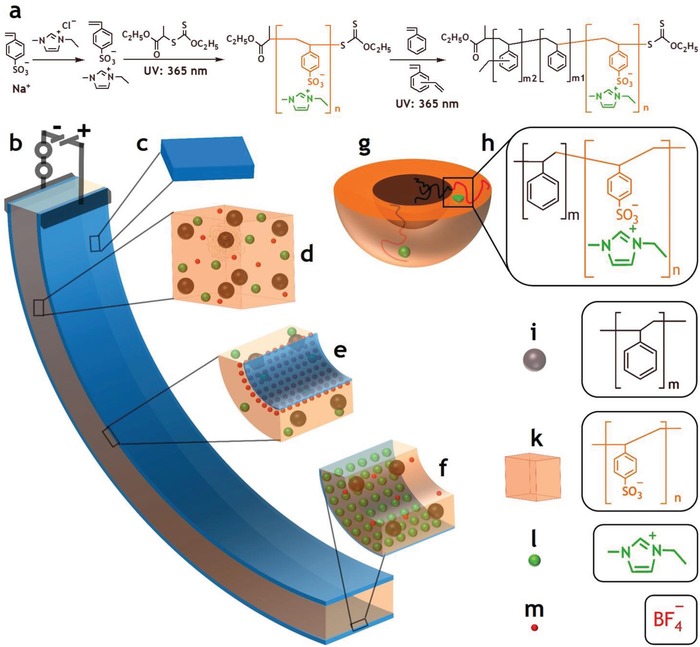
Synthesis of PS‐*b*‐PSS‐EMIm block copolymer for ionic electroactive soft actuators: a) scheme to synthesize the block copolymer, b) activated actuator under a voltage, c) NS codoped graphene/PEDOT:PSS electrode, d) morphology of the block copolymer integrated with EMImBF_4_ ionic liquid, e) anode area, f) cathode area, g) PS‐*b*‐PSS‐EMIm core–shell particle, h) PS‐*b*‐PSS‐EMIm, i) PS sphere, k) PSS matrix, l) EMIm cation, and m) BF_4_^−^ anion.

Although the design of the chemical architecture and morphology of PS‐*b*‐PSS‐EMIm has emerged from our profound interdisciplinary knowledge in the fields of ionic soft actuators and polymers, the practical synthesis of this polymer constitutes a great challenge due to the totally different solubility of the two blocks (PSS‐EMIM dissolves in extremely high polar solvents like water, whereas PS dissolves in nearly nonpolar solvents like toluene). We herein present a simple but effective procedure that permits to realize the designed block copolymer and predetermine the sphere morphology simultaneously (Figure 1a). First, we prepared the monomer, styrene sulfonate 1‐ethyl‐3‐methylimidazolium, by the reaction of the two corresponding salts and characterized it by proton nuclear magnetic resonance (^1^H NMR) spectroscopy (**Figure** **2**a and Figure S2, Supporting Information). Second, for the synthesis of the first block, PSS‐EMIm, we used reversible addition fragmentation chain transfer (RAFT) polymerization initiated by UV light. This method afforded high molecular weight copolymers, which could contribute to the mechanical stability.^5^, ^12^, ^16^ Unlike conventional RAFT polymerization, in which initiators and RAFT agents are different chemicals, xanthate serves both functions in the present polymerization (Figure S3, Supporting Information).^16^ Upon UV irradiation, the xanthate photoinitiator splits into free radicals, which react with the monomer to form growing polymer chains. After the UV light is removed, most of the chains retain the stable photoinitiator end groups, which subsequently react with other monomers to form block copolymers. The PSS‐EMIm polymer was characterized by ^1^H NMR and gel permeation chromatography (GPC), and the corresponding results are included in Figure 2a,b. As shown in Figure 2a, the ^1^H NMR results indicate that the peaks attributed to the EMIm cation remained unaltered after the polymerization, whereas the peaks corresponding to the styrene sulfonate monomer disappeared and were replaced by those of polystyrene sulfonate. From the GPC result (Figure 2b), it was observed that PSS‐EMIm had a high number average molecular weight of 145 000 g mol^−1^ and a low polydispersity index of 1.8, despite such high molecular weight and the low transfer coefficient of xanthate (0.67).^17^ Finally, we added the second block, PS, by emulsion RAFT poly‐merization, which afforded cross‐linked PS spheres. In order to introduce selectively cross‐linkers within the PS spheres, styrene was mixed with an appropriate amount of divinyl benzene before the polymerization. This not only enhances the mechanical stability without sacrificing the conductivity but also prevents such spheres from changing, and consequently allows determining the morphology of the membranes beforehand. Furthermore, this approach enables us to control the morphology over a thick film, which could be inaccessible by using common techniques such as solution casting or solvent annealing. Again, in contrast to conventional emulsion RAFT polymerization, in which macro‐RAFT agents, initiators, and surfactants are separate chemicals, PSS‐EMIm serves all three functions.^18^ The photoinitiating end group absorbs UV light and generates free radicals that react with the styrene monomer, thereby functioning as both macro‐RAFT agent and initiator. On the other hand, due to the presence of the sulfonate groups, PSS‐EMIm dissolves in water and acts as a surfactant, forming shells that cover the growing PS cores and prevent their aggregation. Therefore, the vast difference in the solubility of the two blocks, which was initially a challenge for the synthesis of PS‐*b*‐PSS‐EMIm, became an advantage when the suitable synthetic procedure was used. Furthermore, since additional surfactants were not needed, the as‐prepared polymer was able to be used directly in the next steps without purification. The PS‐*b*‐PSS‐EMIm core–shell structure was then investigated by double aberration‐corrected transmission electron microscopy (Cs‐TEM) as shown in Figure 2d–h. In order to study the core–shell structure of an individual particle, the preparation of a very dilute sample was required due to the easy combination of the outer shells of these core–shell particles (**Figure** **3**a–c). In TEM scanning, high‐angle annular dark‐field (HAADF) imaging is highly sensitive to the variations of atomic numbers in samples, with a higher atomic number resulting in a brighter image.^19^ The HAADF image in Figure 2d shows a clear contrast between the dark core and the bright shell of the block copolymer particle. This result demonstrates that the shell is formed by sulfur‐containing PSS‐EMIm, whereas the core is constituted of PS, which contains mainly carbon. Such core–shell structure was also confirmed by elemental mapping (Figure 2e–h) as oxygen, sulfur, and nitrogen in PSS‐EMIm spread densely in the shell, while carbon distributes regularly in the whole particle. Taken together, these results confirm that, following the appropriate synthetic procedure, the designed block copolymer with sphere morphology could be successfully synthesized.

**Figure 2:**
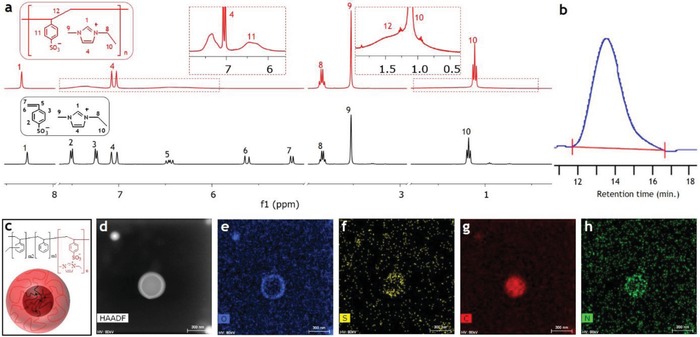
Synthesis of PS‐*b*‐PSS‐EMIm block copolymer core–shell structure: a) ^1^H NMR and b) GPC trace of PSS‐EMIm; c) illustration, d) HAADF TEM; e) oxygen, f) sulfur, g) carbon, and h) nitrogen elemental mapping images of the core–shell structure.

**Figure 3:**
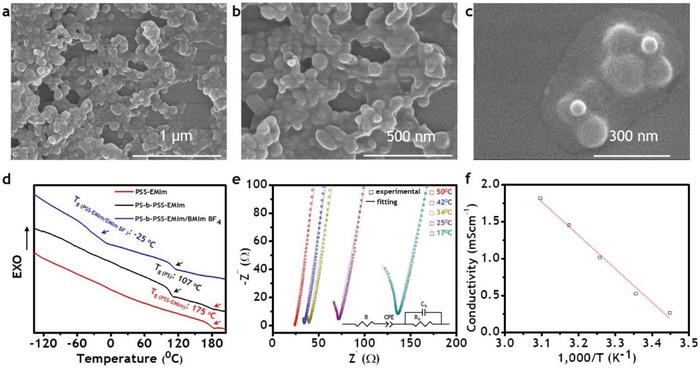
Some properties of PS‐*b*‐PSS‐EMIm block copolymer membrane: a–c) FE‐SEM images, d) DSC curves, e) EIS spectra, and f) ionic conductivity.

Figure 3 summarizes some properties of the PS‐*b*‐PSS‐EMIm membrane. The core–shell particles produced during the synthesis enabled the easy fabrication of electrolyte membranes with continuous conducting phases. Thus, the PSS‐EMIm shells were found to mix to each other naturally as demonstrated in the field emission scanning electron microscopy (FE‐SEM) images in Figure 3a–c, which shows that the shells of these particles were totally combined and densely packed. As can be seen in Figure 3c, even for few particles, their shells merged to form a continuous shell covering all cores. The PS cores, in turn, could remain stable while casting the films due to the cross‐linkers. As a result, despite having a thickness up to 80 micrometers, the corresponding films should exhibit the expected sphere morphology with irregular order due to the high size polydispersity of the PS cores (Figure 3c). For the fabrication of electrolyte membranes, polymers are usually mixed with an ionic liquid. In the case of ionic soft actuators, the ionic liquid plasticizes the polymers, enhancing the ionic conductivity and the size difference between cation and anion contributes to the bending. In addition, ionic liquids with negligible vapor pressure and hydrophobicity allow actuators to be operated under electric stimuli in open‐air atmosphere.^4^, ^8^, ^15^, ^20^ In the present work, we selected 1‐ethyl‐3‐methylimidazolium tetrafluoroborate (EMImBF_4_) since the ionic liquid has the same cation as that of the conducting block, which would facilitate the mixing with our polymer. Moreover, its high conductivity (14.1 mS cm^−1^ at 25 °C) and large size difference between cation (115.53 Å^3^) and anion (54.20 Å^3^) were expected to contribute significantly to large bending deformations under electrical fields. When mixing EMImBF_4_ with PS‐*b*‐PSS‐EMIm, the ionic liquid incorporated exclusively within the PSS‐EMIm conducting block, which caused the *T*_g_ of this block to decrease sharply from 175 to −25 °C, whereas the *T*_g_ of the PS mechanical structure block was kept at 107 °C as evident by the differential scanning calorimetry (DSC) results described in Figure 3d. The low *T*_g_ of the conducting phase implies that the PSS‐EMIm chains became flexible upon being plasticized by EMImBF_4_, which greatly facilitated ion movement. The *T*_g_ slightly above 100 °C for the PS phase stems most likely from the cross‐linkers, which favors the mechanical stability. The samples prepared in this work exhibited a compromising cross‐linking density. The presence of PS phase in PS‐*b*‐PSS‐EMIm block copolymer significantly enhanced the mechanical stability of the polymer membrane containing the ionic liquid, since the PSS‐EMIm homopolymer integrated with EMImBF_4_ became gels and did not form a free‐standing film. Therefore, the polymer membrane would be expected to consist of a continuous flexible (low *T*_g_) conducting phase and a separate strong (high *T*_g_) mechanical structure phase, which could result in high ionic conductivity.^15^ To confirm this, the as‐produced polymer electrolyte membranes were subjected to electrochemical impedance spectroscopy (EIS) analysis in a wide range of temperature, and the results are depicted in Figure 3e,f. Accordingly, a nonconducting membrane in the absence of the ionic liquid became a good conducting membrane with EMImBF_4_ (0.51 mS cm^−1^ at 25 °C, Figure 3f). These results indicate that the ionic conductivity was properly controlled while maintaining a good mechanical stability, thereby confirming the successful design and synthesis of the functionally antagonistic core–shell block copolymer for the specific use in the ionic soft actuators herein described.

Next, in order to fabricate dry‐type actuators, a polymer electrolyte membrane was sandwiched between two electrodes based on nitrogen and sulfur codoped (NS codoped) graphene and poly(3,4‐ethylenedioxythiophene)‐poly(styrenesulfonate) (PEDOT:PSS) by hot pressing (see the Supporting Information for experimental details and Figure S4 in the Supporting Information for an illustration).^7^, ^8^, ^9^ The NS codoped graphene was prepared according to the literature, and its properties are presented in Figure S5 in the Supporting Information.^8^ The optical images of the polymer membrane, electrode, and the as‐prepared actuator are described in Figure S6a–c in the Supporting Information. In order to demonstrate the excellent quality of the as‐prepared block copolymer electrolyte, the performance of the actuator was intended to investigate under ultralow input voltages in open air at ambient conditions, and the results are summarized in **Figure** **4** and Figure S7 in the Supporting Information. As can be seen in Figure 4a,b, under the excitations as low as 0.5 V at 0.1 Hz of frequency, the actuator showed high tip displacements, about 8.2 mm (0.37% strain) for the square input and 6.8 mm (0.32% strain) for the harmonic input. Additionally, the actuator with the length of 15 mm was activated with peak voltage of 1 V and excitation frequency of 0.1 Hz. As shown in Figure S7a in the Supporting Information, the tip displacements (strains) for square and harmonic inputs were 11.3 mm (0.67% strain) and 8.1 mm (0.56% strain), respectively. In order to check the rise time and behavior of the actuator over prolonged periods of time, the displacements were measured under a wide range of ultralow direct current (DC) inputs from 0.1 to 0.5 V, and the results are depicted in Figure 4c. The step responses with much higher DC voltages (0.6–1 V) and longer time over 35 min were shown in Figure S7b in the Supporting Information. Accordingly, the actuator exhibited large, fast, and consistent bending without back relaxation over 35 min, which could be of great benefit to a large range of applications including soft robotics and precise control systems, since such low voltages would be very safe and such fast response without back relaxation would increase accuracy and reliability. The displacements and corresponding strains decreased according to voltage amplitudes, and at the peak voltage of 0.5 V these values were determined to be 4.1 mm and 0.21%, respectively. Higher strains could be obtained by increasing applied voltages and/or thickness, and reducing length of actuators. For example, as shown in Figure S7b in the Supporting Information, the shorter actuator with the length of 15 mm showed much higher strain of 0.46% under the input voltage of 1 V. The inset graph of Figure 4c reveals that the actuator exhibited a very fast response, with rise times as short as 5 s and independent on stimulus amplitudes. Such rise times are slightly shorter than that of Nafion (6 s) and even 12 times and 120 times smaller than those of the aforementioned diblock copolymer and pentablock copolymer, respectively, although the operating voltage amplitudes of the actuator based on PS‐*b*‐PSS‐EMIm were by far lower than that of Nafion and those of the related block copolymers.^4^, ^6^, ^7^ This good performance arises from the high ionic conductivity of the well‐connected continuous conducting phase. Although the present actuator showed significant reduction in rise time, such value of 5 s is still too long for some applications. However, this performance can be greatly enhanced by using highly conductive and capacitive electrodes.^4^, ^8^ In order to test the response of the actuator under different conditions, it was activated by square and sine input voltages with the amplitudes ranging from 0.1 to 0.5 V and frequencies from 0.1 to 5.0 Hz (Figure 4d,e). The tip displacements according to input voltages ranging from 0.05 to 1 V are shown in Figure S7a in the Supporting Information. The performances summarized in Figure 4d,e and Figure S7a in the Supporting Information show that the actuator was able to significantly deform over a wide range of such conditions, which included the very low voltage of 0.05 V and the very high frequency of 5.0 Hz. These results support its great potential for many applications especially soft robotics. The excellent performances are indicative of the high quality of the developed block copolymer electrolyte and show that it is perfectly suitable for the development of ionic soft actuators. As can be extracted from Figure 4d,e, the performance decreased when reducing the voltage amplitudes or increasing the frequencies, which can be attributed to the decline of the electrical field. At a given distance, the electrical field is proportional to the electrical potential. Therefore, lower stimulus amplitudes result in a reduction of the performance. The excitation frequency is directly interrelated with the scan rate that affects capacitance. With a given capacitor, high frequency and high scan rate lead to low capacitance. As depicted in Figure S5l,m in the Supporting Information, the specific capacitance decreased significantly from 190 to 115 F g^−1^ when increasing the frequencies from 0.1 to 5.0 Hz, causing a drop in actuation performance from 8.22 to 0.38 mm. In order to check the cyclic stability of the actuator, it was subjected to the continuous stimulation of 0.5 V and 1 Hz over 4 h, as shown in Figure 4f. It was found that the actuator maintained 94% of its original performance after 14 000 cycles, demonstrating good stability and durability that could be valuable for the aforementioned applications, since these properties ensure an accurate and reliable operation. The optical images of the actuator operating at ±0.5 and ±1.0 V are depicted in Figure S6d,e in the Supporting Information, respectively. Blocking forces generated by the actuator are shown in Figure S7c in the Supporting Information. Accordingly, at 2 V, the actuator was able to generate the force three times higher than its weight. The blocking forces can be greatly enhanced considering mechanical stiffness and electrical conductivity of the actuator itself by mainly increasing the thickness of the polymer electrolyte and using the much stronger electrode such as a carbon fiber woven fabric in the future.

**Figure 4:**
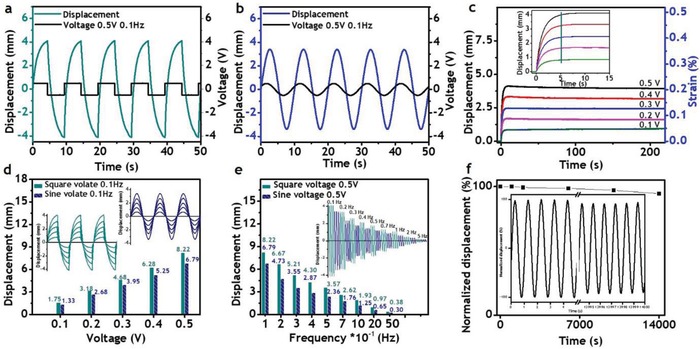
Actuation performance: displacements at 0.5 V and 0.1 Hz under a) square and b) sine voltages; c) displacements according to DC voltages; displacements according to d) voltages, and e) frequencies; and f) durability. (The free length of the actuator was 20 mm.)

In conclusion, we have described the innovative design of a functionally antagonistic core–shell polymer electrolyte with the PS‐*b*‐PSS‐EMIm block copolymer, which is suitable for use in electroactive ionic polymer actuators when synthesized with the appropriate procedure. Such synthetic procedure enabled to not only predetermine the sphere morphology in which the mechanical structure PS block distributes in the continuous conducting PSS‐EMIm matrix, but also regulate the ionic conductivity and the mechanical stability, separately. Therefore, the inversely correlated properties of the copolymer were enhanced simultaneously by plasticizing exclusively the PSS‐EMIm conducting block and introducing selectively cross‐linkers within the PS mechanical structure block. Hence, the block copolymer electrolyte membrane with good ionic conductivity and mechanical stability could be obtained. The actuator prepared from this polymer showed good performance over a wide range of frequencies at ultralow input voltages below 0.5 V. The rise times of the as‐prepared actuator were much shorter than those based on Nafion and the other reported block copolymers. At the ultralow stimulus of 0.5 V, the actuator exhibited high displacement of 8.2 mm (0.37% strain), a very fast rise time within 5 s, and excellent stability and durability over 14 000 cycles. These results demonstrate the success of our approach for the development of the new polymer electrolytes and the present ionic soft actuator thereof as a promising candidate for many applications, especially soft robots. In addition, we believe that this approach could also be extended to the preparation of new polymer electrolytes for other related fields, namely solid‐state lithium battery and fuel cells.

## Conflict of Interest

The authors declare no conflict of interest.

## Supporting information

Supplementary
