# Supplementary material for: Electroactive Artificial Muscles Based on Functionally Antagonistic Core–Shell Polymer Electrolyte Derived from PS‐b‐PSS Block Copolymer
Source: Adv Sci (Weinh). 2018 Dec 13;6(5):1801196. doi: 10.1002/advs.201801196 (PMC6402454; doi:10.1002/advs.201801196)
Supplement: Supplementary file 1 — Supplementary [file ADVS-6-1801196-s001.pdf]

Copyright WILEY-VCH Verlag GmbH & Co. KGaA, 69469 Weinheim, Germany, 2018.

ADVANCED SCIENCE
Open Access

Supporting Information

for *Adv. Sci.,* DOI: 10.1002/advs.201801196

Electroactive Artificial Muscles Based on Functionally Antagonistic Core–Shell Polymer Electrolyte Derived from PS-*b*-PSS Block Copolymer

*Van Hiep Nguyen, Jaehwan Kim, Rassoul Tabassian, Moumita Kotal, Kiwoo Jun, Jung-Hwan Oh, Ji-Myeong Son, Muhammad Taha Manzoor, Kwang Jin Kim, and Il-Kwon Oh**

Copyright WILEY-VCH Verlag GmbH & Co. KGaA, 69469 Weinheim, Germany, 2016.

Supporting Information

Electroactive Artificial Muscles Based on Functionally Antagonistic Core-Shell Polymer Electrolyte Derived from PS-b-PSS Block Copolymer

Van Hiep Nguyen[1], Jaehwan Kim[1], Rassoul Tabassian[1], Moumita Kotal[1], Kiwoo Jun[1], Jung-Hwan Oh[1], Ji-Myeong Son[1], Muhammad Taha Manzoor[1], Kwang Jin Kim[2], and Il-Kwon Oh[1,*]

[1] Prof. I.-K. Oh, V. H. Nguyen, J. Kim, R. Tabassian, M. Kotal, K. Jun, J. H. Oh, J. M. Son, M. T. Manzoor
Creative Research Initiative Center for Functionally Antagonistic Nano-Engineering, Department of Mechanical Engineering, Korea Advanced Institute of Science and Technology (KAIST), 291 Daehak-ro, Yuseong-gu, Daejeon 34141, Republic of Korea

[2] Prof. K. Kim
Active Materials and Smart Living Laboratory, Department of Mechanical Engineering, University of Nevada, Las Vegas (UNLV), Las Vegas, NV, 89154, USA

*Corresponding email should be addressed to ikoh@kaist.ac.kr

Keywords: soft actuator; ionic polymer actuator; polymer electrolyte; block copolymer; soft robotics

Materials: Sodium 4-vinylbenzenesulfonate (SSNa, Sigma-Aldrich, 90%), 1-ethyl-3-methylimidazolium chloride (EMImCl, Io.li.tec., 98%), 1-ethyl-3-methylimidazolium tetrafluoroborate ($EMImBF_4$, Io.li.tec., 98%), ethyl 2-bromopropionate (Sigma-Aldrich, 99%), potassium ethyl xanthogenate (Sigma-Aldrich, 96%), poly(3,4-ethylenedioxythiophene)-poly(styrenesulfonate) (PEDOT:PSS CLEVIOS PH 1000, Heraeus), and some solvents were used as received. Styrene (Junsei, 93%) and divinylbenzene (Sigma-Aldrich, 80%) were purified by passing through alumina (Acros, basic activated type) columns before use.

Synthesis of S-2-ethyl propionate-o-ethyl xanthate: According to a literature procedure, 10.14 g (63.3 mmol) potassium ethyl xanthogenate was mixed with 10.20 g (56.3 mmol) ethyl 2-bromopropionate in 100 mL ethanol at 0 °C under a nitrogen atmosphere in a 250-mL round-bottom flask equipped with a magnetic stirring bar and wrapped in aluminum foil.[S1] After allowing to warm to room temperature (25 °C), the mixture was stirred for 16 hours. Water (100 mL) was then added, and the product was extracted by a mixture of diethyl ether and pentane (1:2 by volume). This step was repeated several times to purify the product. Removal of the solvents resulted in a yellow-green oil, which was dried at room temperature in a vacuum oven. ^1H NMR (400 MHz, $CDCl_3$, δ): 4.58 (q, J = 7.13 Hz, 2H; $C(S)OCH_2$), 4.32 (q, J = 7.38 Hz, 1H; CH), 4.15 (q, J = 7.12 Hz, 2H; $C(O)OCH_2$), 1.52 (d, J = 7.42 Hz, 3H; CH_3CH), 1.37 (t, J = 7.13 Hz, 3H; $C(S)OCH_2CH_3$), 1.24 (t, J = 7.15 Hz, 3H; $C(O)OCH_2CH_3$) (**Figure S3**).

Synthesis of 1-ethyl-3-methylimidazolium 4-styrenesulfonate (SS-EMIm) monomer: 1.7 g (8.2 mmol) SSNa was dissolved in 40 ml dimethylformamide (DMF) in a 100-mL round-bottom flask equipped with a magnetic stirring bar and wrapped in aluminum foil. A solution of 1.3 g (9.2 mmol) EMImCl in 30 ml DMF was then added dropwise. The mixture was

stirred overnight at 25 °C. Removal of sodium chloride and DMF resulted in a crude oil, which was purified by extracting with acetone several times to afford a colorless oil in 76% yield. ^{1}H NMR (400 MHz, D_2O, δ): 8.28 (s, 1H; NCHN), 7.50 (d, J = 8.28 Hz, 2H; Ar H), 7.24 (d, J = 8.08 Hz, 2H; Ar H), 7.05 (d, J = 22.50 Hz, 2H; NCHCHN), 6.46 (q, J = 17.67; 10.94 Hz, 1H; ArCH=CH_2), 5.63 (d, J = 17.74 Hz, 1H; CH=CH_2 trans), 5.14 (d, J = 10.83 Hz, 1H; CH=CH_2 cis), 3.83 (q, J = 7.27 Hz, 2H; NCH_2CH_3), 3.52 (s, 3H; NCH_3), 1.17 (t, J = 7.37 Hz, 3H; CH_2CH_3) (**Figure S2**).

Synthesis of poly(styrene)-block-poly(1-ethyl-3-methylimidazolium 4-styrenesulfonate) (PS-b-PSS-EMIm): PS-b-PSS-EMIm block copolymer was synthesized in two steps via RAFT polymerization using UV irradiation with the wavelength of 365 nm (**Figure 1a**). In the first step, the PSS-EMIm block was prepared. Typically, the exact amount of precursors, SS-EMIm monomer (0.88 g, 1.5 mmol), S-2-ethyl propionate-o-ethyl xanthate (50 μL of a 0.056 M solution in DMF), and milli-Q water (2 mL) were added to a 5-mL ampoule equipped with a magnetic stirring bar. The solution was subsequently degassed via six freeze–pump–thaw cycles before sealing the ampoule with a flame. The solution was then stirred and exposed to the UV lamp at room temperature over a designed period of time. Finally, the PSS-EMIm polymer was diluted with water and precipitated in 1 L cold acetone three times before drying overnight in a vacuum oven at 40 °C. In the second step, PS-b-PSS-EMIm was prepared. Separately, PSS-EMIm (60 mg) was dissolved in milli-Q water (3 mL) for one hour, and styrene (80 μL) was mixed with divinylbenzene (3 μL). The two solutions were transferred to a 5-mL ampoule equipped with a magnetic stirring bar. After degassing the mixture via six freeze–pump–thaw cycles, the ampoule was sealed with a flame. The mixture was stirred and irradiated by the UV lamp over a favorable period of time. Finally, the mixture was stirred in an open space for one day to evaporate the remaining monomers.

Characterization of PS-b-PSS-EMIm: PSS-EMIm was characterized via ^{1}H NMR (400 MHz, D_2O) and gel permeation chromatography (GPC), using a methanol solution 30% in water as an eluent and poly(ethylene oxide)s as standard polymers. A PS-b-PSS-EMIm emulsion was diluted with 2-propanol and subjected to field emission scanning electron microscopy (FE-SEM, Hitachi, S-4800, 10 kV) and double aberration-corrected transmission electron microscopy (Cs-TEM, Field Electron and Ion Company, Titan cubed G2 60-300, 80 kV) analysis to check its morphology. The glass transition temperatures (T_g) of the block copolymer with and without mixing with $EMImBF_4$ ionic liquid were determined via differential scanning calorimetry (DSC 204 F1, NETZSCH). The conductivity of PS-b-PSS-EMIm integrated with $EMImBF_4$ was measured according to the literature.[S2] Accordingly, a sample with a dimension of 10 mm x 10 mm x 0.35 mm was tested over the frequency range from 2,250,000 to 100 Hz and 0.1 V of amplitude. The modulus and ultimate tensile strength of PS-b-PSS-EMIm were measured via a machine by Shimadzu Corporation (model: AGS-10kN) with the crosshead speed of 1 mm/min.

Actuator fabrication: For the preparation of the electrode films, nitrogen and sulfur co-doped (NS co-doped) graphene was synthesized according to the literature (**Figure S5**).[S3] This graphene was dispersed in an aqueous PEDOT:PSS solution containing 5 vol% DMF for 12 hours at room temperature, and the resulting mixture was cast in a Teflon mold at 80 °C until a dry electrode film was formed. Then, the mixture of the block copolymer emulsion with $EMImBF_4$ ionic liquid was cast in a Teflon mold in an oven at 50 °C. When a dry film was formed, the temperature was increased to 80 °C and vacuum was applied for two days, affording a free-standing electrolyte membrane. Finally, the membrane was sandwiched between the two electrodes via hot pressing (**Figure S4**). The actuator was cut into the desired

size to check the performance by an experimental setup containing a laser displacement sensor (Keyence, LK-031), a current amplifier (UPM1504), and a NI-PXI data acquisition system (NI-PXI 1042Q, PXI 6252 board). The strain of the actuator was calculated via the following equation based on the classical Euler-Bernoulli beam mechanics with small bending prerequisite:

$$\varepsilon = \kappa t = \frac{2t\delta}{l^2 + \delta^2}$$

where κ, *t*, *δ*, and l are the bending curvature, the thickness, the displacement, and the free length of the actuator, respectively.[S4]

Table S1. Some properties of electroactive polymer (EAP), shape memory alloy (SMA), and electroactive ceramic (EAC) actuators.[S5]

Properties	Units	EAP	SMA	EAC
Actuation strain	%	> 0.5 (bending strain for ionic polymer actuator) > 300 (in-plane strain for dielectric elastomer actuator)	< 8.0	0.1 – 0.3
Force	MPA	0.1 – 40.0	200.0	30.0 – 40.0
Reaction speed	-	μsec. to min.	μsec. to min.	μsec. to sec.
Density	g/cc	1.0 – 2.5	5.0 – 6.0	6.0 – 8.0
Drive voltage	-	1.0 – 7.0 V (ionic EAP) 10.0 – 150.0 V/μm (electronic EAP)	5.0 V	50.0 – 800.0 V
Consumed power	-	m-Watts	Watts	Watts
Fracture behavior	-	Resilient, elastic	Resilient, elastic	Fragile

Table S2. Some advantages and disadvantages of ionic and electronic EAP actuators.[S5]

Actuators	Advantages	Disadvantages
Ionic EAP	- Produce large displacements<br>- Require low voltages (to 0.05 V in this research)<br>- Produce bi-directional actuation depended on the voltage polarity	- Do not hold strains under DC voltages (except for this research)<br>- Generate slow responses<br>- Induce a relatively low blocking forces<br>- Difficult to produce a consistent materials<br>- Need electrolytes and encapsulations<br>- Low electromechanical coupling efficiency
Electronic EAP	- Operate in room conditions for a long time<br>- Generate rapid responses<br>- Hold strain under DC voltages<br>- Induce relatively large blocking forces	- Require high voltages (150 MV/m)<br>- Require compromise between strain and stress<br>- Inadequate for low-temperature beyond T_g and high temperature beyond Curie point<br>- Produce monopolar actuation independent of voltage polarity

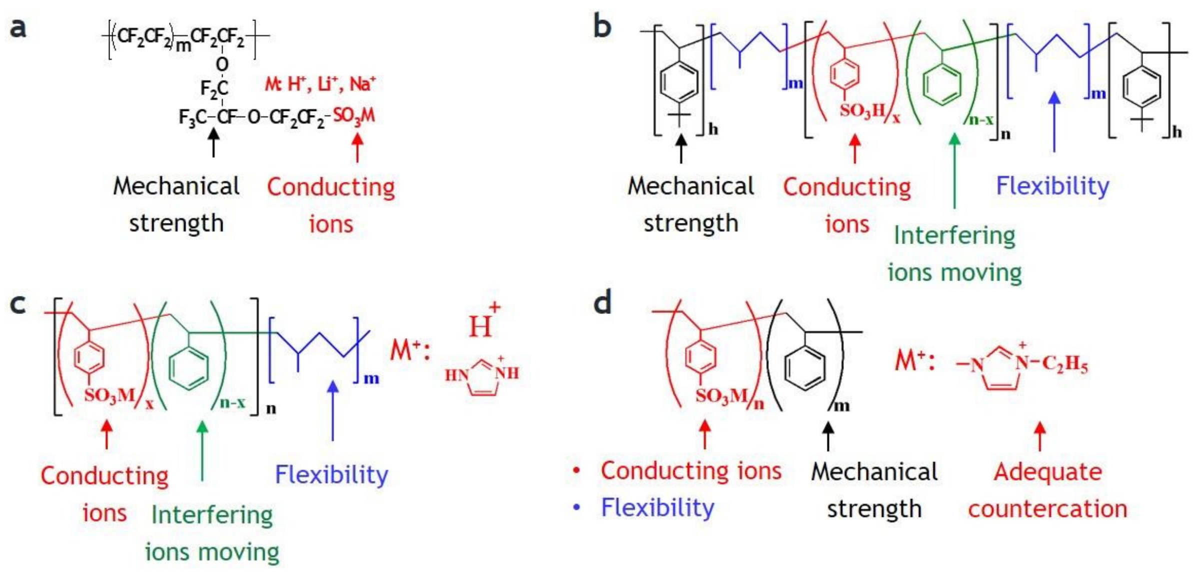

Figure S1. Some typical polymers used in ionic actuators: a) Nafion, b) pentablock copolymer, c) PSS-PMB block copolymer, d) PS-b-PSS-EMIm, the herein presented block copolymer.[S6]

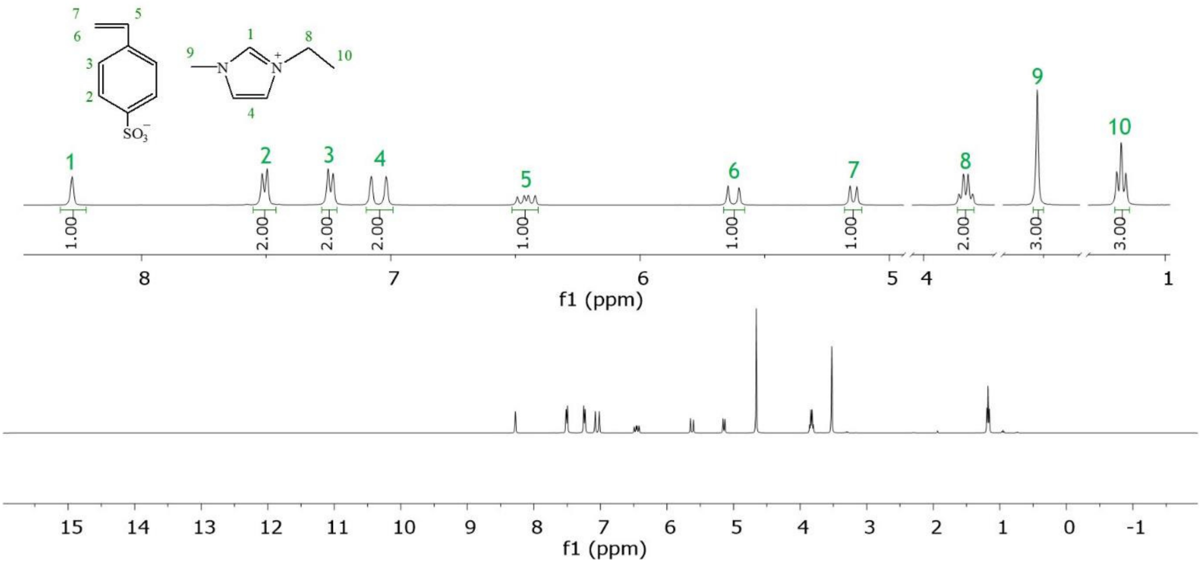

Figure S2. ^{1}H NMR spectrum of SS-EMIm monomer.

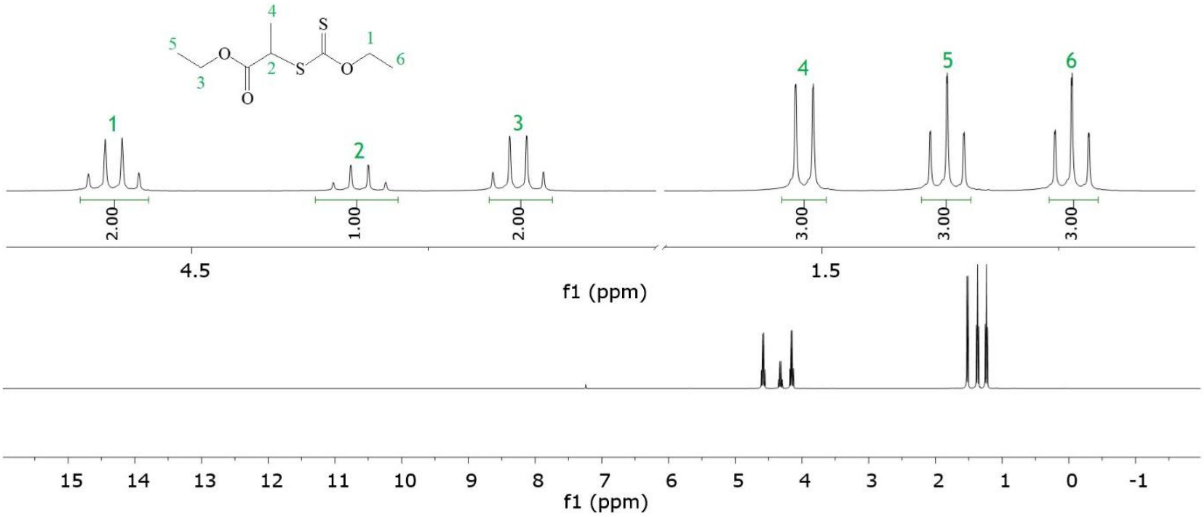

Figure S3. ^{1}H NMR spectrum of s-2-ethyl propionate-O-ethyl xanthate.

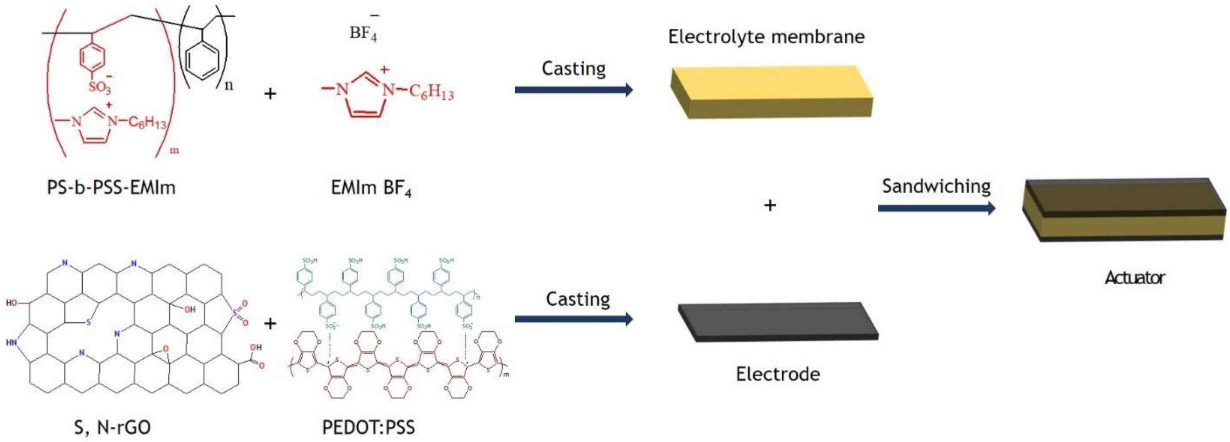

Figure S4. Fabrication of ionic soft actuator using PS-b-PSS-EMIm/EMImBF$_4$ electrolyte membrane and NS co-doped graphene/PEDOT:PSS electrodes.

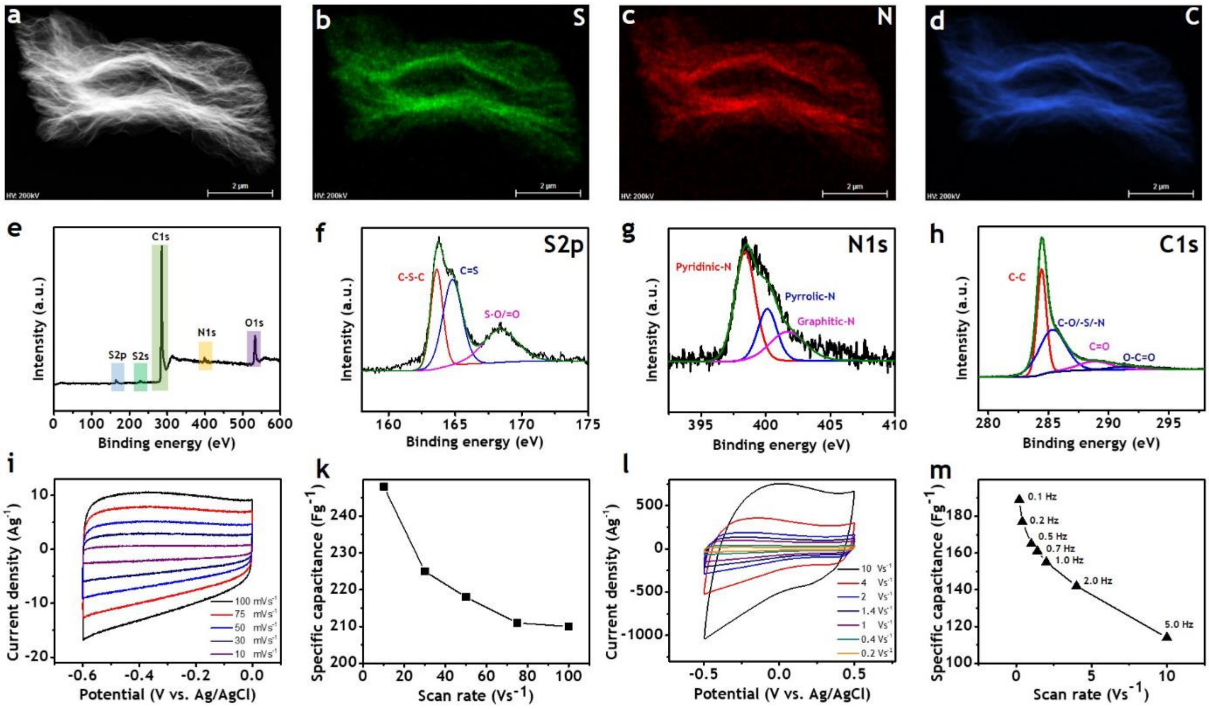

Figure S5. NS co-doped graphene for electrodes: a) TEM image and the corresponding b) sulfur, c) nitrogen, and d) carbon elemental mapping images. e) XPS survey spectra, f) S2p, g) N1s, and h) C1s. Cyclic voltammetry analysis at different scan rates: i) in the potential window from -0.6 to 0 V and k) the corresponding specific capacitance, and l) in the potential window from -0.5 to 0.5 V and m) the corresponding specific capacitance.

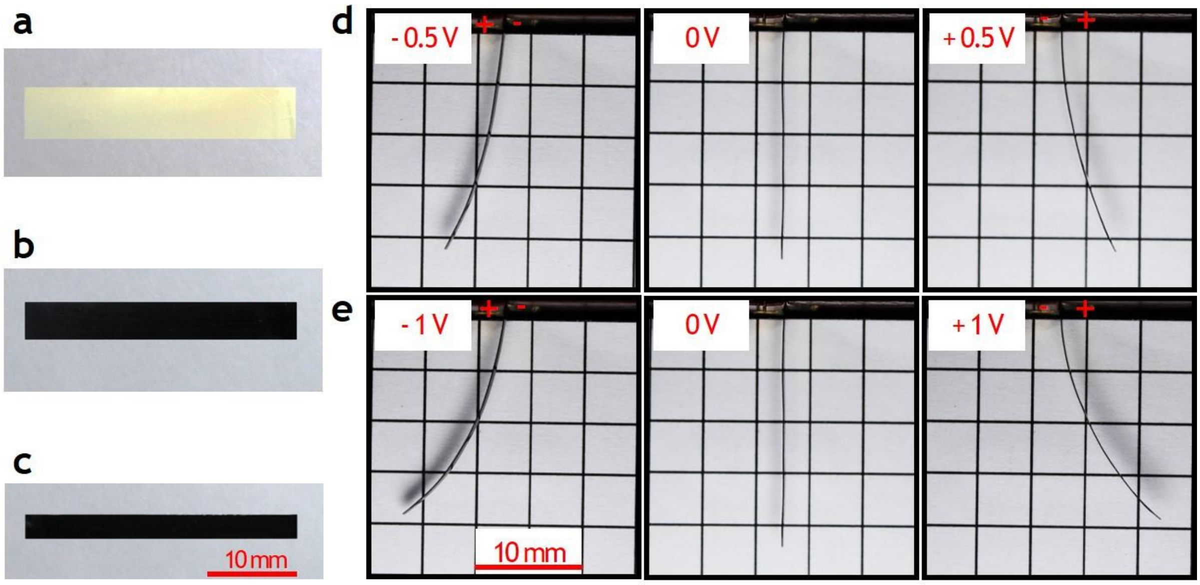

Figure S6. Optical images: a) PS-b-PSS-EMIm / EMIm - BF_4 membrane, b) NS co-doped graphene / PEDOT:PSS electrode, c) actuator; actuation displacements at d) 0.5 V and e) 1 V.

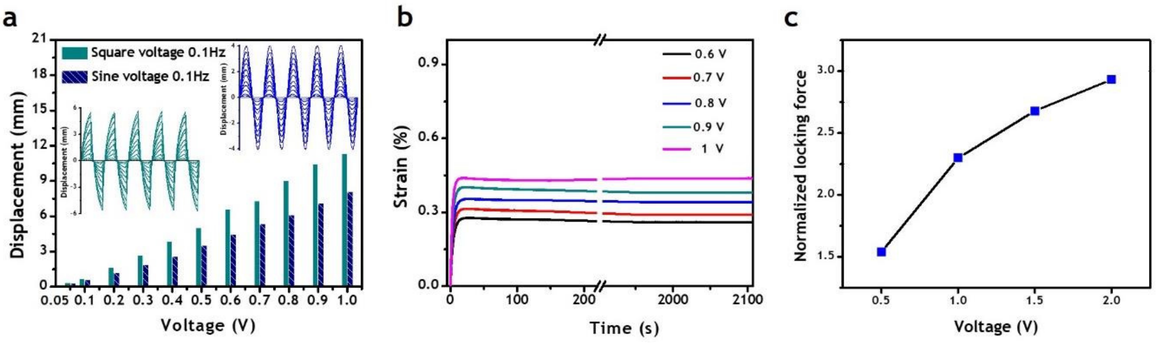

Figure S7. Performance validation of the actuator that has not been operated with electrical stimuli for nine months: a) tip displacements according to square and harmonic input voltages, b) strain according to direct current (DC) voltage over 35 minutes, and c) mormalized blocking force comparing to the weight of the actuator. (The length of the actuator used in this result is 15 mm).

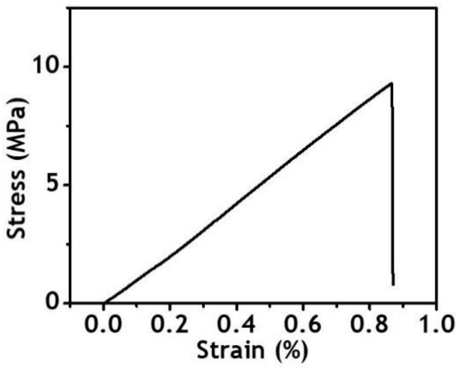

Figure S8. Stress-strain curve of PS-b-PSS-EMIm. (Young modulus is 1.06 GPa and ultimate tensile strength 9.3 MPa.)

Reference

[S1] M. Destarac, C. Brochon, J. M. Catala, A. Wilczewska, S. Z. Zard, Macromol. Chem. Phys. **2002,** 203, 2281.

[S2] T. Soboleva, Z. Xie, Z. Shi, E. Tsang, T. Navessin, S. Holdcroft, J. Electroanal. Chem. **2008,** 622, 145.

[S3] M. Kotal, J. Kim, K. J. Kim, I. K. Oh, Adv. Mater. **2016,** 28, 1610.

[S4] A. Punning, V. Vunder, I. Must, U. Johanson, G. Anbarjafari, A. Aabloo, J. Intell. Material Syst. Struct.**2016**, 27(8), 1061.

[S5] Y. Bar-Cohen, Electroactive polymer (EAP) actuators as artificial muscles: reality, potential, and challenges, SPIE – The International Society of Optical Engineering, 2004.

[S6] a) C. Jo, D. Pugal, I. K. Oh, K. J. Kim, K. Asaka, Prog. Polym. Sci. **2013**, 38, 1037; b) O. Kim, T. J. Shin, M. J. Park, Nat. Commun. **2013,** 4, 2208; c) J. W. Lee, S. Yu, S. M. Hong, C. M. Koo, J. Mater. Chem. C **2013,** 1, 3784; d) O. Kim, T. J. Shin, M. J. Park, Nat. Commun. **2016,** 7, 13576.
